# Oral Health Status as a Mediating Effect Between Psychosocial Factors and Oral Health-Related Quality of Life

**DOI:** 10.1016/j.identj.2026.109514

**Published:** 2026-03-19

**Authors:** Linxin Jiang, Natalie Loschke, Dirk Ziebolz, Daniel R. Reissmann, Gerhard Schmalz

**Affiliations:** a Department of Prosthodontics, University of Leipzig, Leipzig, Germany; b Department of Conservative Dentistry and Periodontology, Brandenburg Medical School Theodor Fontane (MHB), Brandenburg/Havel, Germany; c Department of General Medicine, Leipzig University, Leipzig, Germany

**Keywords:** Oral health-related quality of life, Self-efficacy, Social support, Oral health, Linear analysis

## Abstract

**Introduction and aims:**

While oral health status and psychosocial factors are associated with oral health-related quality of life (OHRQoL), the mechanisms remain unclear. This study aims to investigate their relationships and explore potential mediating effects.

**Methods:**

Cross-sectional data were collected from dental examination and psychosocial scales of patients who visited the dental clinic for routine check-ups and/or preventive measures in 2023. All data were analysed using SPSS with the AMOS plugin. Data preprocessing included Little’s MCAR test and multiple imputation. Reliability and validity tests were conducted to optimize the psychosocial scales. The Mann-Whitney U test and Spearman test analysed the differences and correlations between variables. A hierarchical multiple linear regression identified factors associated with the OHRQoL score and identified potential mediating effects. The structural equation model (SEM) supplementarily quantified the mediating effects.

**Results:**

104 adults, with a median age of 59 years, were included. The linear regression results were consistent with statistical mediation by oral health status (periodontal treatment need, plaque control record (PCR), caries treatment need), such that the direct association between psychosocial scores (self-efficacy of tooth-brushing score (SEoTB), self-efficacy of inter-dental cleaning (SEoIDC)) and the OHRQoL score was no longer evident after inclusion of oral health variables. The bias-corrected and accelerated (BCa) 95% confidence interval for the mediating path was −0.102 and −0.011 (*P* < .05). The mediating effect was −0.055, accounting for 12.6% of the total effect.

**Conclusions:**

Higher oral hygiene-related self-efficacy (OHRSE) is associated with better OHRoL, with this association partly explained by more favourable clinical oral health status.

**Clinical Relevance:**

These results highlight the potential of combining psychological and oral health management strategies in both individualized treatment and public health programs. By strengthening oral hygiene self-efficacy, clinicians may reduce treatment needs, improve periodontal and caries status, and consequently enhance OHRQoL.

## Introduction

Oral health-related quality of life (OHRQoL) reflects an individual’s subjective evaluation of how oral health affects daily functioning and well-being.^1^ It encompasses dental and periodontal status as well as psychosocial aspects such as self-awareness and social interaction.^2^^,^^3^ OHRQoL has been recognized as an important part of overall health by the World Health Organization.^4^ Given its critical role in evaluating interventions and informing public health programs, understanding the factors associated with OHRQoL has become an important area of investigation.

Many factors influence OHRQoL, including oral hygiene habits (such as oral cleanliness) and oral conditions (such as gum, tooth, mucosa, and denture).^5^ Psychosocial factors such as social support (SS) and self-efficacy (SE) also play a critical role in influencing OHRQoL, alongside traditional clinical and demographic factors.^6^ These psychosocial factors are generally considered important upstream determinants of health and are extensively integrated into the biopsychosocial model for disease management.^7^, ^8^, ^9^, ^10^ Specifically, SS refers to the emotional and practical support received from the social network, which can buffer stress and promote healthy behaviors.^11^ SE, such as general SE and oral hygiene-related self-efficacy (OHRSE), refers to confidence in the ability to execute behaviours required to achieve a desired outcome (eg, maintaining good oral hygiene).^12^ There is growing evidence that higher levels of social support and self-efficacy are associated with better OHRQoL.^6^^,^^13^^,^^14^

However, evidence on the association between psychosocial factors and OHRQoL remains limited, as most studies examined only direct associations.^15^, ^16^, ^17^ Therefore, this study aims to address this gap by exploring the relationship between psychosocial factors (SS, SE, and OHRSE) and OHRQoL in adults. Hierarchical multiple linear regression was used to assess the impact of those factors on OHRQoL and structural equation modelling (SEM) was employed for exploratory analysis. This study will help clarify the mechanisms by which psychosocial factors influence OHRQoL, providing a theoretical foundation for future oral health interventions and management. The study hypothesizes that psychosocial factors enhance OHRQoL by improving oral health status.

## Methods

### Study sample

This cross-sectional study examined psychosocial factors and oral health conditions. It was part of a comprehensive assessment of medical history of patients attending the dental clinic and was approved by the Ethics Committee of the medical faculty of Leipzig University (No. 487/20-ek). All participants were informed verbally and in writing about the study and provided written informed consent for participation.

Participants were consecutively recruited, with a target sample size of at least 100. This target was defined pragmatically (feasibility-based) rather than derived from a formal a priori power calculation. The minimum target of ≥100 was chosen to support stable estimation in the planned multivariable analyses.

Inclusion criteria were that patients should be regularly attending the dental clinic of Leipzig University, be at least 18 years of age and provided written informed consent for the use of anonymized data. The following exclusion criteria were: active prosthodontic therapy or temporary restorations, acute dental problems, severe general diseases making an oral examination and dental therapy impossible, cognitive disorders, inability to answer the questions (eg, due to insufficient oral health knowledge) and pregnancy.

### Measures

All participants provided written informed consent. Dental examination and psychosocial scales (SS, SE, OHRSE, OHRQoL) were collected.

#### Dental examination

A Florida probe was used to examine 28 teeth (excluding third molars) under artificial lighting. The examination included periodontal, caries, and prosthetic status. Specifically, periodontal status was evaluated based on the plaque control record (PCR), gingival bleeding index (GBI), and history of periodontal and maintenance treatment. The periodontal treatment need was determined using the Periodontal Screening Index, participants with a code 3 in at least two sextants or a code 4 were classified as periodontal treatment need. Caries status was evaluated based on the decayed, missing, and filled teeth (DMFT) index. A tooth was coded as “decayed” if the dental examination identified visible cavities, notable breakdown beneath the enamel, or clearly detectable softened areas. The presence of decayed teeth was used to define caries treatment need. Prosthetic status was evaluated based on the presence of dental implants, removable partial dentures, or fixed partial dentures.

#### SS score

The assessment of social support was conducted using the short form of the social support questionnaire (F-SozU-K14), which comprises 14 items.^18^ Responses were recorded ranging from 1 (disagree) to 5 (fully agree). Higher total scores indicate greater level of social support.

#### SE score

The assessment of self-efficacy was conducted using the scale developed by Schwarzer and Jerusalem, which comprises 10 items.^19^ Responses were recorded ranging from 1 (disagree) to 4 (fully agree). Higher total scores indicate greater levels of self-efficacy.

#### OHRSE score

The assessment of OHRSE was conducted using the dental self-efficacy scale, which comprises 19 items.^20^ The scale assessed three dimensions: the self-efficacy of tooth-brushing (SEoTB) score, consisting of 6 items; the self-efficacy of inter-dental cleaning (SEoIDC) score, comprising 6 items; and the self-efficacy of dental visits (SEoDV) score, comprising 7 items. Responses were recorded ranging from 1 (completely confident not to) to 4 (completely confident to). Higher total scores indicate greater OHRSE.

#### OHRQoL

The assessment of OHRQoL was conducted using the German short form of the Oral Health Impact Profile (OHIP-G5), comprising 5 items.^21^ The scale assessed four dimensions: oral function (items 1 and 2), orofacial pain (item 3), orofacial appearance (item 4), and psychosocial impact (item 5). Responses were recorded ranging from 0 (never) to 4 (very often). Higher total scores indicate worse OHRQoL.

### Variable categories design

The variables were categorized into three groups. The first category was demographic factors, including gender (male, female), smoking status (no, yes), and age. The second category was psychosocial scores, including SS score, SE score, SEoTB score, SEoIDC score, and SEoDV score. The third category was oral health status, including periodontal treatment need (no, yes), PCR, GBI, periodontal treatment (no, yes), maintenance treatment (no, yes), caries treatment need (no, yes), decayed, missing, and filled teeth (DMFT), and dental prosthesis (no, yes).

### Statistical analysis

All data analyses were performed using IBM SPSS Statistics (version 25.0) and integrated AMOS plugin (version 26.0). Data visualization was conducted using GraphPad Prism (version 8.0.2). Continuous variables were described using the median and interquartile range (IQR). Categorical variables were described with number (*N*) and percentage (%).

During the data preprocessing, Little’s MCAR test was conducted (*χ*^2^ = 121.072, *df* = 513, *P* > .05), indicating that the data met the criteria for missing completely at random (MCAR). Multiple imputation was applied to handle missing values, and the dataset with the highest Cronbach’s α was retained for subsequent analysis. The reliability and validity of scales were assessed using Cronbach’s α test and confirmatory factor analysis (CFA). Acceptable reliability was defined as Cronbach’s α and composite reliability (CR) > 0.7, and acceptable validity was defined as outer loading and average variance extracted (AVE) > 0.5. To optimize the scales’ structure, items that did not meet those criteria were removed.^22^^,^^23^

The Kolmogorov–Smirnov test found that all continuous variables were non-normally distributed. Thus, the Mann–Whitney U test was used for differences between two independent groups, and Spearman’s rank correlation was used for the correlation between continuous variables. Variables with *P* < .20 were included in the subsequent hierarchical multiple linear regression to avoid prematurely excluding potentially important predictors or confounders.^24^^,^^25^

The linear regression explored the associated factors of the OHRQoL score and identified potential mediating effects. A Box-Cox transformation was performed on the outcome variable to correct for skewness before conducting regression analysis. Block 1 included demographic factors, Block 2 incorporated psychosocial scores, and Block 3 incorporated oral health status. A decrease in the standardized regression coefficient (β) or the loss of statistical significance upon the addition of subsequent blocks indicated the presence of a mediating effect, respectively. Model fit was evaluated using the coefficient of determination (R^2^) and adjusted R^2^, which represent the proportion of variance explained by the model. Multicollinearity was considered acceptable when the variance inflation factor (VIF) was <5.

A SEM was constructed as a supplementary analysis to support the regression-based findings and to provide an additional check of the indirect pathway. The mediating effect = (the β of the independent variable on the mediator) * (the β of the mediator on the dependent variable). The proportion of mediation = the ratio of the mediating effect/the total effect. Statistical significance of the mediating path was confirmed when the bias-corrected and accelerated (BCa) 95% confidence interval (CI) from 5000 bootstrap resamples did not include zero. Model fit was considered acceptable when the Chi-square (*χ*^2^)/the degrees of freedom (*df*) was ≤2, the root mean square error of approximation (RMSEA) was <0.09, and the comparative fit index (CFI) was >0.90.

A two-tailed *P* < .05 was considered statistically significant.

## Results

### Descriptive analysis of demographics and psychosocial scores

To achieve acceptable reliability and validity, one item from the SS scale, five items from the SE scale, and one item from the OHRQoL scale were removed (Table S1).

As shown in Table 1, a total of 104 participants were included, with 31.7% male and 68.3% female. The median age was 59 years (IQR: 50.0-68.6). The median scores for psychosocial measures were as follows: SS score, 60.5 (total score: 65); SE score, 15.0 (total score: 20.0); SEoTB score, 24.0 (total score: 24.0); SEoIDC score, 20.0 (total score: 24.0); SEoDV score, 25.0 (total score: 28.0); and OHRQoL score, 1.0 (total score: 20.0).

**Table 1:** Demographic information and psychosocial scores of participants.

Variables	*N* (%)	Median (IQR)
Total	104 (100.0)	
Gender		
Male	33 (31.7)	
Female	71 (68.3)	
Age (years)		59.0 (50.0-68.6)
SS score		60.5 (56.0-64.0)
SE score		15.0 (14.0-17.0)
SEoTB score		24.0 (22.0-24.0)
SEoIDC score		20.0 (16.0-24.0)
SEoDV score		25.0 (23.0-28.0)
OHRQoL score		1.0 (0.0-3.0)

IQR, Interquartile range; OHRQoL, oral health-related quality of life; SE, self-efficacy; SEoTB, self-efficacy of tooth-brushing; SEoIDC, self-efficacy of inter-dental cleaning; SEoDV, self-efficacy of dental visit; SS, social support.

### Univariate analysis

The analysis of differences in OHRQoL score among different groups and the correlation between variables (Table 2) showed that participants with periodontal treatment need, caries treatment need, or dental prosthesis had higher median of OHRQoL scores (*P* < .05). SEoTB score and SEoIDC score were negatively correlated with OHRQoL score (*P* < .05), and GBI was positively correlated with OHRQoL score (*P* < .05).

**Table 2:** Differences and correlations analysis of OHRQoL and related factors.

Categories	Variables	OHRQoL score (Median (IQR))	Statistical value	*P*
Demographic Factors	Gender		−1.636*	.102
	Male	0.0 (0.0-2.0)		
	Female	2.0 (0.0-3.0)		
	Smoking		−0.303*	.762
	No	1.0 (0.0-3.0)		
	Yes	0.0 (0.0-5.0)		
	Age (years)		0.028†	.775
Psychosocial scores	SS score		0.046†	.640
	SE score		−0.061†	.538
	SEoTB score		−0.280†	**.004^⁎⁎^**
	SEoIDC score		−0.309†	**.009^⁎⁎^**
	SEoDV score		−0.127†	.198
Oral health status	Periodontal treatment need		−2.100*	.036*
	No	0.0 (0.0-3.0)		
	Yes	2.0 (2.0-4.5)		
	PCR		0.184†	.062
	GBI		0.254†	**.009^⁎⁎^**
	Periodontal treatment		−0.621*	.535
	No	0.5 (0.0-3.0)		
	Yes	1.5 (0.0-3.0)		
	Maintenance treatment		−1.054*	.292
	No	0.5 (0.0-3.0)		
	Yes	1.5 (0.0-3.0)		
	Caries treatment need		−2.078*	.038*
	No	0.0 (0.0-3.0)		
	Yes	2.0 (0.0-4.3)		
	DMFT		0.142†	.150
	Dental prosthesis		−2.273*	.023*
	No			
	Yes			

DMFT, decayed, missing, and filled teeth index; GBI, gingival bleeding index; OHRQoL, oral health-related quality of life; PCR, plaque control record; SE, self-efficacy; SEoTB, self-efficacy of tooth-brushing; SEoIDC, self-efficacy of inter-dental cleaning; SEoDV, self-efficacy of dental visit; SS, social support.

Note: *P < .05 and ^⁎⁎^*P* < .01 are highlighted in bold.

⁎ U value of Mann-Whitney U test.

† Spearman’s correlation coefficient*.*

### Hierarchical multiple linear regression analysis

In the univariate analysis, variables with *P* < .20, including gender, SEoTB score, SEoIDC score, SEoDV, periodontal treatment need, PCR, GBI, caries treatment need, DMFT, and dental prosthesis were incorporated into the linear regression.

As presented in Table 3, the regression analysis showed that the progressive inclusion of different blocks of category enhanced the model’s ability to explain the variance in OHRQoL score. Following the addition of oral health status in Block 3, the model accounted for 21.3% of the variance (*P* < .05). Periodontal treatment need, GBI, and caries treatment need were positively associated with OHRQoL score (*P* < .05). Compared with Block 2, the regression coefficients (B) of SEoTB score and SEoIDC score were attenuated and became non-significant. This suggests that no remaining direct association was evident once oral health variables were included in the model.

**Table 3:** Hierarchical multiple linear regression model of OHRQoL and related factors.

Categories	Variables	R^2^ (R^2^ change)	B	se	β	*P*	VIF
Block 1		0.034 (0.024)					
Demographic Factors	Gender						
	Male†		-	-	-	-	-
	Female		−1.011	0.537	−0.183	.062	1.000
Block 2		0.074 (0.037)					
Demographic Factors	Gender						
	Male†		-	-	-	-	-
	Female		−1.072	0.544	−0.195	.051	1.041
Psychosocial scores	SEoTB score		−0.035	0.016	−0.247	.037*	1.452
	SEoIDC score		−0.161	0.073	−0.258	.030*	1.501
	SEoDV score		−0.029	0.056	−0.052	.610	1.104
Block 3		**0.213 (0.128)**					
Demographic Factors	Gender						
	Male†		-	-	-	-	-
	Female		−0.801	0.539	−0.145	.141	1.129
Psychosocial scores	SEoTB score		−0.031	0.016	−0.217	.062	1.502
	SEoIDC score		−0.138	0.070	−0.221	.052	1.554
	SEoDV score		−0.053	0.056	−0.095	.349	1.195
Oral health status	Periodontal treat need						
	No†		-	-	-	-	-
	Yes		1.085	0.515	0.210	.038*	0.901
	PCR		0.001	0.009	0.012	.909	1.412
	GBI		0.030	0.012	0.242	.013*	1.467
	Caries treat need						
	No†		-	-	-	-	-
	Yes		1.070	0.502	0.207	.036*	1.199
	DMFT		0.003	0.039	0.009	.931	1.355
	Dental prosthesis						
	No†		-	-	-	-	-
	Yes		1.209	0.710	0.186	.092	1.406

R^2^, the coefficient of determination; B, unstandardized regression coefficient; se, standard error; β, standardized regression coefficient; DMFT, decayed, missing, and filled teeth index; GBI, gingival bleeding index; OHRQoL, oral health-related quality of life; PCR, plaque control record; SE, self-efficacy; SEoTB, self-efficacy of tooth-brushing; SEoIDC, self-efficacy of inter-dental cleaning; SEoDV, self-efficacy of dental visit; SS, social support; VIF, variance inflation factor.

Note: The R**^2^** results of ANOVA test with *P* < .05 are highlighted in bold.

† Reference variable.

⁎ *P* < .05 is highlighted in bold*.*

### SEM of the mediating effect

The psychosocial scores (SEoTB score, SEoIDC score) were set as an independent variable, the oral health status (periodontal treatment need, GBI, and caries treatment need) was set as a mediator, and the OHRQoL score was set as a dependent variable. The SEM showed a good model fit (*χ*^2^/*df* = 0.941, CFI = 1.000, RMSEA = 0.000, GFI = 0.956), as shown in Figure.

**Figure:**
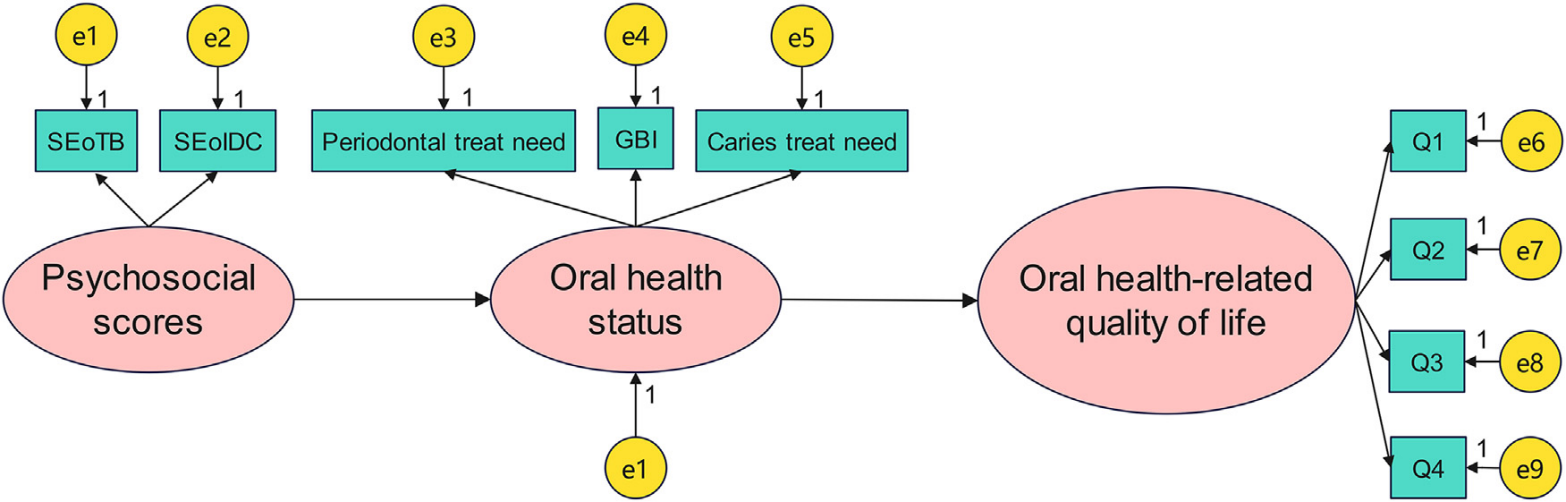
The SEM for the mediating effect path. *P < .05. e, error term; GBI, gingival bleeding index; OHRQoL, oral health-related quality of life; SEoTB, self-efficacy of tooth-brushing; SEoIDC, self-efficacy of inter-dental cleaning.

Path analysis indicated that the psychosocial scores were negatively associated with oral health status (β_1_ = −0.125, *P* = .042), and oral health status was positively associated with the OHRQoL score (β_2_ = 0.437, *P* = .015). Thus, the mediating effect was −0.055 (β_1_ * β_2_). Bootstrap resampling further confirmed the significance of the mediating path of oral health status (BCa 95% CI: −0.102 to −0.011, *P* < .05). In linear regression analysis, the total effect of psychosocial scores on the OHRQoL score was −0.438, with SEoTB score (β = −0.217) and SEoIDC score (β = −0.221). Accordingly, the proportion of the mediating effect through oral health status accounted for 12.6% (−0.055/−0.438), suggesting that, indicating that the indirect pathway via oral health status explained a share of the overall association in this cross-sectional analysis.

## Discussion

This study suggests that oral health status may represent a key pathway linking psychosocial factors to OHRQoL, highlighting the potential value of strengthening these psychosocial dimensions within oral health management.

Some previous studies found that higher levels of social support and self-efficacy were positively associated with OHRQoL.^13^^,^^26^^,^^27^ Additionally, SE had a mediating effect on the association between SS and OHRQoL.^28^ However, this study did not identify a significant association between SS, SE, and OHRQoL. There are several potential explanations for this finding. First, the characteristics of participants should be considered. Participants were recruited from a dental clinic where individuals sought treatment, preventive care, or routine examinations. Thus, OHRQoL may have been primarily driven by oral health status, potentially overshadowing the impact of other general psychosocial factors. This context may also restrict variability in broader psychosocial resources and shift perceived impacts toward immediate clinical needs and symptoms, thereby attenuating associations with more distal constructs such as SS and SE. In addition, the low median OHIP-G5 score in this study suggests a potential floor effect, which can constrain outcome variability and attenuate effect estimates, particularly for more distal psychosocial constructs.^29^ Second, although validated instruments were used, a small number of items were removed based on prespecified psychometric criteria in this sample. Such data-driven refinement may reduce content coverage and limit direct comparability with studies using the original scale forms. Therefore, these findings should be interpreted with caution and replicated in larger samples using the full, original instruments. Third, the null findings for SS and SE may indicate that broad psychosocial resources exert context-dependent relationships with OHRQoL compared with oral hygiene–related self-efficacy, which is more closely tied to oral health behaviours and clinical status. In addition, prior evidence suggests that the relationship between SS and OHRQoL may vary by population and context (eg, older or socially vulnerable groups), which could partly explain inconsistencies across studies.^30^

Periodontal and caries treatment needs were key predictors of OHRQoL in this study. The mechanisms can be explained from several perspectives. First, periodontal diseases and caries contribute to pain and discomfort.^31^^,^^32^ Additionally, periodontal diseases affect self-esteem.^33^ Furthermore, gingivitis has been found to impose a functional and aesthetic burden.^34^ Those factors all contribute to the negative impact on OHRQoL. Those findings support the notion that oral health status, including periodontal and caries status, are critical determinants of OHRQoL, emphasizing the necessity of oral health interventions to enhance OHRQoL. From a clinical and public health perspective: patients with greater periodontal or caries treatment needs and poorer oral hygiene indicators may benefit most from timely clinical management combined with patient-centred behavioural support to reduce oral impacts on daily life.

Current research found that the mechanism through which OHRSE impacts oral health primarily operates via the promotion of oral health behaviors. For instance, a study conducted among adults showed that a higher OHRSE level was associated with increased toothbrushing frequency, which is a crucial preventive measure against periodontal diseases and dental caries.^17^ In addition, previous studies have identified a partial mediating effect of oral health status, indicating that psychosocial factors impact OHRQoL both directly and indirectly.^28^ Unlike general psychosocial factors, OHRSE primarily focuses on the perceived ability to maintain oral hygiene behaviors.^35^ As a result, its impact on OHRQoL was not observed as a direct psychological moderating factor but was instead reflected through improvements in oral health status. However, it is also noteworthy that oral health status may account for only a modest proportion of the overall association in this analysis. Psychological factors may still relate to OHRQoL through pathways that are not fully captured by clinical oral health indicators. For example, dental anxiety and sense of coherence constructs have been reported to be associated with poorer OHRQoL, potentially through heightened perceived burden and avoidance of dental care.^36^ In addition, cognitive–affective responses to symptoms (eg, pain-related anxiety) may amplify the perceived intensity and functional interference of oral discomfort, thereby adversely affecting OHRQoL.^37^ Taken together, these considerations imply that improving OHRQoL may require both clinical treatment of disease burden and targeted psychosocial components, particularly among individuals who report greater oral impacts despite relatively similar clinical status. Overall, future research should explore the integration of psychosocial interventions into oral health management systems to develop more effective strategies. For instance, the integration of psychological interventions into dental treatment could contribute to the improvement of dental care and support the maintenance of oral hygiene habits following periodontal treatment.^27^^,^^38^, ^39^, ^40^

There are several limitations to this study. First, the participants were recruited from a specific clinic, which may limit the generalizability of the findings. Given the modest sample size relative to the number of covariates, smaller effects may have been underpowered and coefficient estimates should be interpreted as exploratory. Thus, larger multicentre studies are warranted to confirm the stability of the models. Second, although reliability and validity assessments were conducted during both the investigation process and data processing stages, the measurements of OHRQoL and psychosocial scores were self-reported, which may be subject to recall and social desirability bias. Third, as a clinical indicator, GBI reflects oral hygiene and periodontal health status in this study. However, the absence of other relevant parameters, such as probing depth and periodontal attachment loss, constitutes a limitation, restricting the clinical applicability of the findings. Lastly, due to the study design, causal relationships cannot be established in this cross-sectional study. To avoid over-interpretation, future studies with larger sample sizes and preferably longitudinal designs are needed to confirm the stability of the SEM results and to further validate the proposed indirect pathway.

## Conclusion

Higher OHRSE is associated with better OHRQoL and this relationship is mediated through having a better oral health status. The integration of comprehensive intervention strategies, particularly those incorporating psychological support related to oral hygiene and oral health management, may contribute to an enhancement of OHRQoL.

## Conflict of interest

The authors declare that they have no known competing financial interests or personal relationships that could have appeared to influence the work reported in this paper.

## Appendix Supplementary materials

Image, application 1
