# Supplementary material for: Oral Health Status as a Mediating Effect Between Psychosocial Factors and Oral Health-Related Quality of Life
Source: Int Dent J. 2026 Mar 19;76(3):109514. doi: 10.1016/j.identj.2026.109514 (PMC13018937; doi:10.1016/j.identj.2026.109514)
Supplement: Supplementary file 1 [file mmc1.docx]

**SUPPLEMENTARY TABLE 1** Construct validity and reliability assessment in the psychosocial scales.

| **Constructs** | **Items** | **Reliability** | | **Validity** | |
| --- | --- | --- | --- | --- | --- |
|  |  | **Cronbach’ α** | **CR** | **AVE** | **Outer loading** |
| **Acceptable threshold** |  | >0.7 | | >0.5 | |
| **SS scale** |  | 0.926 | 0.929 | 0.506 |  |
|  | SS1 |  |  |  | 0.596 |
|  | SS2 |  |  |  | 0.670 |
|  | SS3 |  |  |  | 0.529 |
|  | SS4 |  |  |  | 0.671 |
|  | SS5 |  |  |  | 0.843 |
|  | SS6 |  |  |  | 0.695 |
|  | SS7 |  |  |  | 0.639 |
|  | SS8 |  |  |  | 0.687 |
|  | SS9 |  |  |  | 0.848 |
|  | SS10 |  |  |  | 0.906 |
|  | SS11 |  |  |  | 0.803 |
|  | SS12 |  |  |  | 0.626 |
|  | SS13 |  |  |  | 0.626 |
| **SE scale** |  | 0.845 | 0.847 | 0.528 |  |
|  | SE1 |  |  |  | 0.781 |
|  | SE2 |  |  |  | 0.775 |
|  | SE3 |  |  |  | 0.622 |
|  | SE4 |  |  |  | 0.809 |
|  | SE5 |  |  |  | 0.623 |
| **SEoTB scale** |  | 0.856 | 0.873 | 0.538 |  |
|  | SEoTB1 |  |  |  | 0.616 |
|  | SEoTB2 |  |  |  | 0.712 |
|  | SEoTB3 |  |  |  | 0.840 |
|  | SEoTB4 |  |  |  | 0858 |
|  | SEoTB5 |  |  |  | 0.667 |
|  | SEoTB6 |  |  |  | 0.675 |
| **SEoIDC scale** |  | 0.933 | 0.935 | 0.707 |  |
|  | SEoIDC1 |  |  |  | 0.784 |
|  | SEoIDC2 |  |  |  | 0.777 |
|  | SEoIDC3 |  |  |  | 0.813 |
|  | SEoIDC4 |  |  |  | 0.890 |
|  | SEoIDC5 |  |  |  | 0.875 |
|  | SEoIDC6 |  |  |  | 0.898 |
| **SEoDV scale** |  | 0.889 | 0.896 | 0.555 |  |
|  | SEoDV1 |  |  |  | 0.704 |
|  | SEoDV2 |  |  |  | 0.709 |
|  | SEoDV3 |  |  |  | 0.620 |
|  | SEoDV4 |  |  |  | 0.899 |
|  | SEoDV5 |  |  |  | 0.686 |
|  | SEoDV6 |  |  |  | 0.745 |
|  | SEoDV7 |  |  |  | 0.818 |
| **OHRQoL scale** |  | 0.770 | 0.809 | 0.515 |  |
|  | OHRQoL1 |  |  |  | 0.778 |
|  | OHRQoL2 |  |  |  | 0.750 |
|  | OHRQoL3 |  |  |  | 0.681 |
|  | OHRQoL4 |  |  |  | 0.654 |

**Abbreviations**: CR, composite reliability; AVE, average variance extracted; SS, social support; SE, social efficacy; SEoTB, self-efficacy of tooth-brushing; SEoIDC, self-efficacy of inter-dental cleaning; SEoDV, self-efficacy of dental visit; OHRQoL, oral health-related quality of life.
